# Antigenic and Molecular Characterization of Avian Influenza A(H9N2) Viruses, Bangladesh

**DOI:** 10.3201/eid1909.130336

**Published:** 2013-09

**Authors:** Karthik Shanmuganatham, Mohammed M. Feeroz, Lisa Jones-Engel, Gavin J.D. Smith, Mathieu Fourment, David Walker, Laura McClenaghan, S.M. Rabiul Alam, M. Kamrul Hasan, Patrick Seiler, John Franks, Angie Danner, Subrata Barman, Pamela McKenzie, Scott Krauss, Richard J. Webby, Robert G. Webster

**Affiliations:** St. Jude Children’s Research Hospital, Memphis, Tennessee, USA (K. Shanmuganatham, D. Walker, L. McClenaghan, P. Seiler, J. Franks, A. Danner, S. Barman, P. McKenzie, S. Krauss, R.J. Webby, R.G. Webster);; Jahangirnagar University, Dhaka, Bangladesh (M.M. Feeroz, S.M.R. Alam, M.K. Hasan);; University of Washington, Seattle, Washington, USA (L. Jones-Engel);; Duke-NUS Graduate Medical School, Singapore (G.J.D. Smith, M. Fourment);; Duke Global Health Institute, Durham, North Carolina, USA (G.J.D. Smith)

**Keywords:** influenza, avian influenza A(H9N2) viruses, viruses, H9N2, H5N1, H7N3, molecular characterization, reassortants, Bangladesh, zoonoses, transmission, avian influenza, respiratory infections

## Abstract

Human infection with avian influenza A(H9N2) virus was identified in Bangladesh in 2011. Surveillance for influenza viruses in apparently healthy poultry in live-bird markets in Bangladesh during 2008–2011 showed that subtype H9N2 viruses are isolated year-round, whereas highly pathogenic subtype H5N1 viruses are co-isolated with subtype H9N2 primarily during the winter months. Phylogenetic analysis of the subtype H9N2 viruses showed that they are reassortants possessing 3 gene segments related to subtype H7N3; the remaining gene segments were from the subtype H9N2 G1 clade. We detected no reassortment with subtype H5N1 viruses. Serologic analyses of subtype H9N2 viruses from chickens revealed antigenic conservation, whereas analyses of viruses from quail showed antigenic drift. Molecular analysis showed that multiple mammalian-specific mutations have become fixed in the subtype H9N2 viruses, including changes in the hemagglutinin, matrix, and polymerase proteins. Our results indicate that these viruses could mutate to be transmissible from birds to mammals, including humans.

Initially infecting poultry, avian influenza A(H9N2) viruses have been sporadically identified in pigs and humans, which suggests that some of these viruses have adapted to bind mammalian host receptors or have acquired mutations that increase mammalian receptor specificity (*1*–*3*). Human infection with avian influenza A(H9N2) virus was initially identified in Hong Kong and China in 1999 (*4*); in 2011, infection with this subtype was reported for a patient in Bangladesh (*5*). Detection of these viruses in humans outside of China highlights the necessity and urgency for comprehensive surveillance because of the viruses’ expanding host range.

Phylogenetically, avian influenza A(H9N2) viruses can be grouped into 3 distinct sublineages represented by their prototype strains: A/Qa/HK/G1/97 (G1-like), A/Dk/HK/Y280/97 (Y280-like), and A/Ck/Korea/38349-p96323/96 (Korean-like) (*1*,*6*,*7*). Genetic and antigenic analyses of subtype H9N2 isolates from the past 2 decades have shown that these viruses are gradually evolving from the Eurasian lineage into several distinct sublineages and are becoming established in domestic poultry (*7*–*18*). Phylogenetic analyses of subtype H9N2 viruses isolated in China and the Middle East have shown that these viruses have undergone reassortment with other subtypes to generate multiple novel genotypes consisting of gene segments from different lineages (*7*,*11*,*13*,*19*,*20*). 

Worldwide, Bangladesh is among countries with the highest numbers of reported outbreaks of highly pathogenic avian influenza (HPAI) (H5N1) (*21*). Since an initial outbreak in February 2007, Bangladesh has reported 550 outbreaks of infection with HPAI (H5N1) virus (493 at commercial farms and 57 among backyard poultry) (*22*–*24*). In Bangladesh, live-bird markets are the most common outlets for purchase of poultry and poultry meat; an estimated 95% of poultry meat and eggs sold in the country are sold at these markets (*25*). Previous surveillance conducted at live-bird markets in Bangladesh found that avian influenza virus (AIV) is prevalent (23%); the low pathogenicity H9N2 subtype predominated, but other subtypes were isolated, including H5N1, H1N2, H1N3, H3N6, H4N2, and H10N7 (*26*). However, this surveillance report did not include information about the molecular properties of circulating subtype H9N2 viruses.

We reviewed surveillance data and conducted molecular and genetic analyses of influenza A(H9N2) viruses circulating among poultry in Bangladesh. Our study had 3 primary goals: 1) characterize the antigenic and molecular properties of subtype H9N2 isolates; 2) define genetic and phylogenetic relationships between the genes identified in these viruses and those of other AIVs; and 3) determine whether these viruses have acquired genomic changes that could facilitate transmission from avian to mammalian hosts.

## Materials and Methods

### Sample Collection and Screening

Surveillance for influenza viruses in poultry began in Bangladesh in November 2008. Each month, trained personnel collected 200–600 samples from apparently healthy domestic live birds (chickens, quail, pigeons, ducks, and turkeys) at retail markets, a pet market, chicken layer and duck farms, and wild birds (50–200 samples from each location). Samples consisted of oropharyngeal, cloacal, and environmental (fecal matter and water samples from cages and fecal digesters) swab specimens. 

During November 2008–August 2011, a total of 17,438 samples (3,078 oropharyngeal, 3,377 cloacal, 10,983 environmental) were collected (Table 1). Screening for AIVs was performed as described (*26*). Briefly, all samples were subjected to real-time reverse transcription PCR by using influenza A–specific primer and probes; samples with positive results were injected into egg to confirm the presence or absence of virus. A total of 734 AIV (H9N2) isolates were extracted from the 17,438 samples collected (Table 1).

**Table 1:** Sources and subtypes of avian influenza viruses isolated in Bangladesh, 2008–2011

Site	Primary species from which samples were obtained*	Primary species from which sample was influenza positive	Total no. samples	Subtypes		
				H1N2, H1N3, H3N6, H3N8, H4N2, H10N7	H5N1	H9N2
Live bird market-1	Quail	Quail	821		20	37
Live bird market-2	Chickens, ducks, pigeons	Chickens	1,691	12	13	85
		Duck				2
Live bird market-3	Chickens	Chickens	1,877	7	38	360
		Ducks				2
Live bird markets-4	Chickens	Chickens	46			12
Live bird markets-5	Chickens	Chickens	2,677	14	20	205
		Duck				2
Pet market	Pet and wild birds	Quail	1,859	3	1	20
Farm-1	Ducks	Ducks	100	24		
Farm-2	Chickens	Chickens	3,945	2		9
Farm-3	Chickens		40			
Farm-4	Ducks		100			
Farm-5	Ducks		100			
Farm-6	Ducks		901			
Wild birds, environmental-1	Wild birds		836			
Wild birds, environmental -2	Wild birds		471			
Wild ducks, environmental-1	Water		1,724			
Wild ducks, environmental-2	Ducks		250			
Total			17,438	62	92	734

*Other species that were sampled at some locations included turkeys, wild birds, and exotic birds.

### Virus Isolation and Propagation

To determine the genetic and evolutionary diversity of the AIV (H9N2) viruses circulating in Bangladesh, we selected 44 of the 734 isolates for further analysis (Table 2); these isolates were representative of the location, time, species, and sample types from which they were isolated. With the exception of 1 isolate from a chicken on a farm, all isolates examined were from chicken, duck, and quail samples collected at retail markets. The viruses were propagated in 10-day-old embryonated chicken eggs, and initial subtyping was done by sequencing the hemagglutinin (HA) and neuraminidase (NA) genes, as described (*26*).

**Table 2:** Avian influenza A(H9N2) virus isolates from animals in live bird markets, Bangladesh, 2008–2011*

Isolate	Host common name	Sample type	Mixtures†
A/Env/BD/907/2009 (quail)	Quail	F	NDV
A/Env/BD/1041/2009 (duck)	Duck	F	H5
A/Dk/BD/1231/2009	Duck	OP	H5
A/Ck/BD/2075/2009	Chicken	OP	H5
A/Pigeon/BD/4303/2009	Pigeon	OP	NDV
A/Env/BD/5144/2009 (quail)	Quail	F	H5
A/Ck/BD/5209/2009	Chicken	OP	NDV
A/Env/BD/5745/2010 (duck)	Duck	F	H5
A/Env/BD/8202/2010 (chicken)	Chicken	F	
A/Ck/BD/8411/2010	Chicken	OP	
A/Ck/BD/8413/2010	Chicken	OP	
A/Ck/BD/8415/2010	Chicken	OP	
A/Env/BD/8463/2010 (chicken)	Chicken	W	
A/Env/BD/8465/2010 (chicken)	Chicken	W	
A/Ck/BD/8725/2010	Chicken	OP	
A/Ck/BD/8731/2010	Chicken	OP	
A/Ck/BD/8996/2010	Chicken	C	NDV
A/Ck/BD/9029/2010	Chicken	OP	NDV
A/Env/BD/9306/2010 (parrot)	Parrot	F	NDV
A/Ck/BD/9334/2010	Chicken	OP	
A/Env/BD/9350/2010 (chicken)	Chicken	W	NDV
A/Env/BD/9457/2010 (chicken)	Chicken	F	
A/Env/BD/10234/2011 (chicken)	Chicken	W	
A/Env/BD/10306/2011 (quail)	Quail	F	NDV
A/Env/BD/10307/2011 (quail)	Quail	F	NDV
A/Env/BD/10313/2011 (quail)	Quail	F	
A/Env/BD/10316/2011	Quail	F	
A/Ck/BD/10401/2011	Chicken	OP	
A/Ck/BD/10411/2011	Chicken	OP	
A/Ck/BD/10450/2011	Chicken	C	NDV
A/Ck/BD/10897/2011	Chicken	OP	
A/Ck/BD/11154/2011	Chicken	C	NDV
A/Env/BD/11173/2011 (chicken)	Chicken	F	
A/Ck/BD/11309/2011	Chicken	OP	
A/Ck/BD/11315/2011	Chicken	OP	
A/Env/BD/11597/2011 (chicken)	Chicken	W	
A/Env/BD/12068/2011 (pigeon)	Pigeon	F	NDV
A/Env/BD/12077/2011 (turkey)	Turkey	F	NDV
A/Env/BD/12093/2011 (quail)	Quail	F	
A/Env/BD/12103/2011 (quail)	Quail	F	
A/Env/BD/12116/2011 (quail)	Quail	F	
A/Env/BD/12119/2011 (quail)	Quail	F	
A/Ck/BD/13916/2011	Chicken	OP	H5
A/Ck/BD/13962/2011	Chicken	OP	H5

*F, fecal; NDV, Newcastle disease virus; OP, oropharyngeal; W, water; C, cloacal. †Some hosts infected with influenza (H9N2) viruses were co-infected with H5N1 or NDV; thus, mixtures of isolates were obtained from some birds. Species in parentheses indicate that the samples were collected from the cages where these particular birds were caged.

### Hemagglutination Inhibition Assay

Polyclonal serum samples were obtained from ferrets that had been inoculated with 1 of the following: prototype viruses from different influenza (H9N2) lineages; influenza (H9N2) isolates from chickens or quail collected in Bangladesh; or a prototype human isolate provided by the US Centers for Disease Control and Prevention. We then conducted hemagglutination inhibition (HI) assays for these serum samples by using 0.5% chicken erythrocytes, as described (*27*).

### Phylogenetic Analysis and Molecular Characterization

Viral RNA extraction and reverse transcription PCR were performed as described (*28*,*29*). DNA sequencing using specific primers was completed in the Hartwell Center at St. Jude Children’s Research Hospital (Memphis, TN, USA). Samples were analyzed by using the BigDye Terminator v3.1 Cycle Sequencing Kit (Invitrogen, Carlsbad, CA, USA) on 3730XL DNA analyzers (Applied Biosystems, Foster City, CA, USA), according to the manufacturers’ recommendations. DNA sequences for all genes were edited, compiled, assembled, and analyzed by using SeqMan in Lasergene 8 (DNASTAR, Madison, WI, USA). Nucleotide sequences were compared with sequences available in GenBank. Multiple nucleotide sequence alignment and the alignment of the deduced amino acids of all of the gene segments were conducted by using ClustalW in BioEdit 7.09.0 (www.mbio.ncsu.edu/bioedit/bioedit.html). The phylogeny of each gene was investigated within the maximum-likelihood framework by using PhyML under the general time reversible substitution model with gamma rate heterogeneity (*30*). The robustness of the grouping was assessed by using 100 bootstrapping replicates (*30*). MegAlign in Lasergene 8 (DNASTAR) was used to determine the percentage of nucleotide sequence similarities, and the NetNGlyc 1.0 Server (*31*) was used to predict the glycosylation sites in the HA and NA genes. The sequences we obtained (Technical Appendix, Table 1) were deposited into GenBank under accession nos. KC757782–KC758115. 

## Results

### Prevalence of Avian Influenza in Bangladesh

During November 2008–August 2011, a total of 17,438 samples were collected from live-bird markets, farms, and wild and environmental sources (*26*). In the markets and farms, samples were predominantly collected from chicken, quail, and ducks. The environmental samples were collected from cages in the markets in which only 1 species was housed (e.g., only chickens or only ducks). The wild bird samples were from a lake that was a feeding station for wild migratory ducks. 

The predominant subtypes identified in these samples were low pathogenicity H9N2 (n = 734) and highly pathogenic H5N1 (n = 92) (Table 1). Other influenza subtypes identified, at low percentages and only during 2008–2009, were H1N2, H1N3, H3N6, H3N8, H4N2, and H10N7 (Table 1). None of the wild-bird samples tested and only 35 (0.87%) of 4,045 farm samples had positive test results for avian influenza (Table 1). The subtype H9N2 and H5N1 viruses were isolated from domestic poultry, mainly chickens and quail that were sampled in live-bird or pet markets (Table 1). 

**Figure 1:**
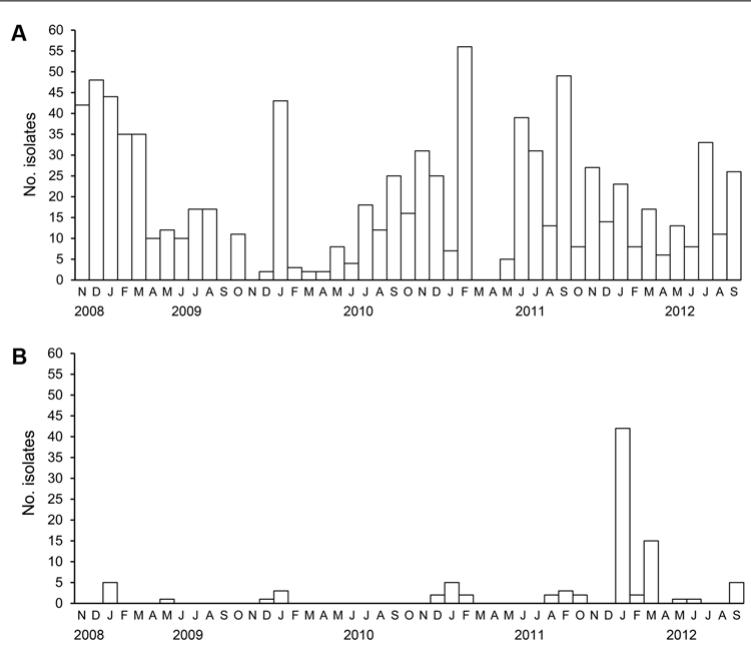
Influenza virus surveillance in Bangladesh, November 2008–September 2012, showing the total number of avian influenza viruses isolated per month. A) Low pathogenicity subtype H9N2 viruses; B) highly pathogenic subtype H5N1 viruses.

The number of subtype H5N1 viruses isolated from field samples increased during the surveillance period (Figure 1, panel B). The numbers of other influenza subtypes identified were low during 2009–2010; during 2010–2011, subtypes H9N2 and H5N1 were most commonly isolated. 

AIVs and Newcastle disease virus (NDV) are the 2 most common viruses that infect poultry in Bangladesh; therefore, all of the influenza-positive samples that were subtyped and used in this study were screened again for H9N2, H5N1, and NDV to determine whether a single host species was co-infected with AIV and NDV. In a substantial proportion of the isolates screened, we identified mixtures of either subtype H9N2 and NDV or subtypes H9N2 and H5N1 (Table 2). All of the samples containing a mixture of viruses were obtained from apparently healthy birds. The highest incidence of influenza (H5N1) virus isolation was during the winter months (January–March), whereas subtype H9N2 was isolated year-round (Figure 1, panel A)

### Antigenic Properties

Antigenic analysis showed that most chicken H9N2 viruses circulating in Bangladesh were antigenically homogenous and similar to the human H9N2 virus (A/BD/0994/11) isolated from Bangladesh (Table 3, Appendix). Antigenic variation was seen in subtype H9N2 viruses isolated from quail; specifically, A/Env/BD/907/2009 (H9N2) (quail) was distinguishable from other contemporary quail isolates when postinfection ferret serum of A/Env/BD/907/2009 (H9N2) (quail) and chicken H9N2 viruses was used (Table 3, Appendix). The chicken H9N2 viruses had moderate cross-reaction to antiserum of A/Env/BD/907/2009 (H9N2) (quail), indicating slight antigenic variation from subtype H9N2 viruses isolated from quail (Table 3, Appendix). The low level of cross-reactivity seen across the quail viruses and between the quail and chicken viruses indicates that the HAs of the subtype H9N2 viruses maintained in quail were antigenically distinct from those of subtype H9N2 viruses maintained in chickens. In addition to the quail isolate, A/Env/BD/12068/2011 (H9N2) (pigeon) reacted with 2 serum samples, the polyclonal serum against A/Env/BD/907/2009 (H9N2) (quail) and that against A/Ck/Pk/(NARC-2434)/06 (H9N2). The HI titers of those reactions were 320 and 160, respectively.

**Table 3:** Antigenic analyses of avian influenza A(H9N2) viruses isolated from poultry in Bangladesh during 2008–2011 compared with reference strains*

Viruses and designations	HI titers† obtained with reference ferret hyperimmune serum directed against											
	G1 clade			Y280 Clade			G1 clade		Bangladesh subtype H9N2 viruses			
	Qa/HK/G1/97	HK/1073/97		Ck/HK/G9/97	Ck/Bei/1/94		Ck/Pk (Swabi)/NARC/2434/06		BD/0994/11	Env/BD/600/08	Env/BD/5473/09	Env/BD/907/09
Reference viruses and designation												
A/Qa/HK/G1/97	160	320		–	–		–		–	–	–	–
A/Ck/HK/G9/97	40	80		640	320		160		160	80	80	–
A/Ck/Beijing/1/94	–	–		–	80		–		–	–	–	–
A/HK/1073/97 (human)	80	320		–	–		–		–	–	–	–
A/Ck/Pk(Swabi)/NARC–2434/06	80	160		320	320		2,560		2,560	2,560	1,280	320
A/BD/0994/2011 (human)	80	160		160	320		1,280		2,560	1,280	640	160
A/Env/BD/600/2008 (chicken)	80	160		160	320		1,280		2,560	1,280	1,280	160
A/Env/BD/907/2009 (quail)	40	80		40	40		80		80	40	–	160
A/Env/BD/5473/2009 (chicken)	80	160		320	320		2,560		2,560	2,560	1,280	640
Bangladesh isolates												
From chickens, n = 27‡												
A/Ck/BD/2075/2009	–	80		160	320		640		2,560	1,280	640	160
A/Ck/BD/5209/2009	40	160		320	320		1,280		2,560	2,560	640	320
A/Env/BD/8202/2010 (chicken)	80	160		320	320		1,280		1,280	1,280	640	320
A/Env/BD/8465/2010 (chicken)	160	320		640	320		2,560		>5,120	2,560	2,560	320
A/Ck/BD/8725/2010	80	160		320	320		1,280		2,560	1,280	640	160
A/Ck/BD/9334/2010	40	80		160	160		640		1,280	640	320	160
A/Env/BD/10234/2011 (chicken)	40	160		320	320		1,280		>5,120	2,560	1,280	320
A/Ck/BD/10450/2011	80	160		320	320		640		1,280	640	1,280	160
A/Ck/BD/11309/2011	40	160		320	320		1,280		2,560	1,280	1,280	320
A/Ck/BD/13962/2011	40	160		160	320		320		640	1,280	320	80
From quail, n = 10‡												
A/Env/BD/5144/ 2009 (quail)	–	40		40	40		160		160	160	80	40
A/Env/BD /10306/2011 (quail)	40	160		40	40		40		40	80	–	–
A/Env/BD/10316/2011 (quail)	160	320		80	80		160		80	80	80	80
A/Env/BD/12103/ 2011 (quail)	40	40		40	40		40		40	–	–	40
A/Env/BD/12116/ 2011 (quail)	–	–		–	–		–		–	–	–	–
A/Env/BD/12119/2011 (quail)	80	160		80	80		80		160	80	80	80
From other poultry species, n = 7‡												
A/Env/BD/1041/2009 (duck)	–	–		–	–		–		–	–	–	–
A/Dk/BD/1231/2009	40	160		160	320		1,280		2,560	1,280	640	320
A/Pi/BD/4303/2009	40	160		160	160		640		2,560	1,280	640	160
A/Env/BD/12068/2011 (pigeon)	160	160		40	80		80		80	40	40	80
A/Env/BD/9306/2010 (parrot)	80	160		160	320		640		2,560	1,280	640	320
A/Env/BD/12077/2011 (turkey)	40	160		320	320		1,280		>5,120	2,560	640	320

*Boldface indicates homologous serum. HI, hemagglutination inhibition.  †HI tests were performed according to standard procedures (*27*).Titers are expressed as the reciprocal of the highest dilution of the last dilution that completely inhibited hemagglutination of 0.5% chicken erythrocytes. ‡Total number of influenza (H9N2) isolates with a similar HI pattern.

These antigenic analyses show that antigenic variation occurs among influenza (H9N2) viruses isolated from different host species. The AIV (H9N2) viruses from Bangladesh cross-reacted well to antiserum against G1 clade H9N2 viruses that were isolated in Bangladesh, Dubai, or Pakistan; however, they showed low reactivity to antiserum against A/Qa/HK/G1/97 (prototype G1), indicating that the viruses from Bangladesh were related to but very distinct from the G1 clade isolates. These results indicate antigenic diversity within the G1 clade.

### Phylogenetic Relationships

The phylogenetic relationships of all 8 genes of the 44 representative AIV (H9N2) viruses were analyzed on the basis of their nucleotide sequences and by comparing them with nucleotide sequences of subtype H9N2 viruses belonging to the main lineages from Asia (*14*). All sequences from Bangladesh except 1 formed a monophyletic cluster. Phylogenetic analyses showed that all the subtype H9N2 viruses isolated from Bangladesh are part of the lineage represented by A/Qa/HK/G1/97 (G1), which is the most dominant lineage worldwide. The HA, NA, nucleoprotein (NP), matrix (M), and polymerase PB2 genes of the viruses from Bangladesh originated from the prototype G1 virus A/Qa/HK/G1/97 (H9N2). The nonstructural (NS) and polymerase PA and PB1 genes were closely related to those in the subtype H7N3 isolate from Pakistan (*14*).

**Figure 2:**
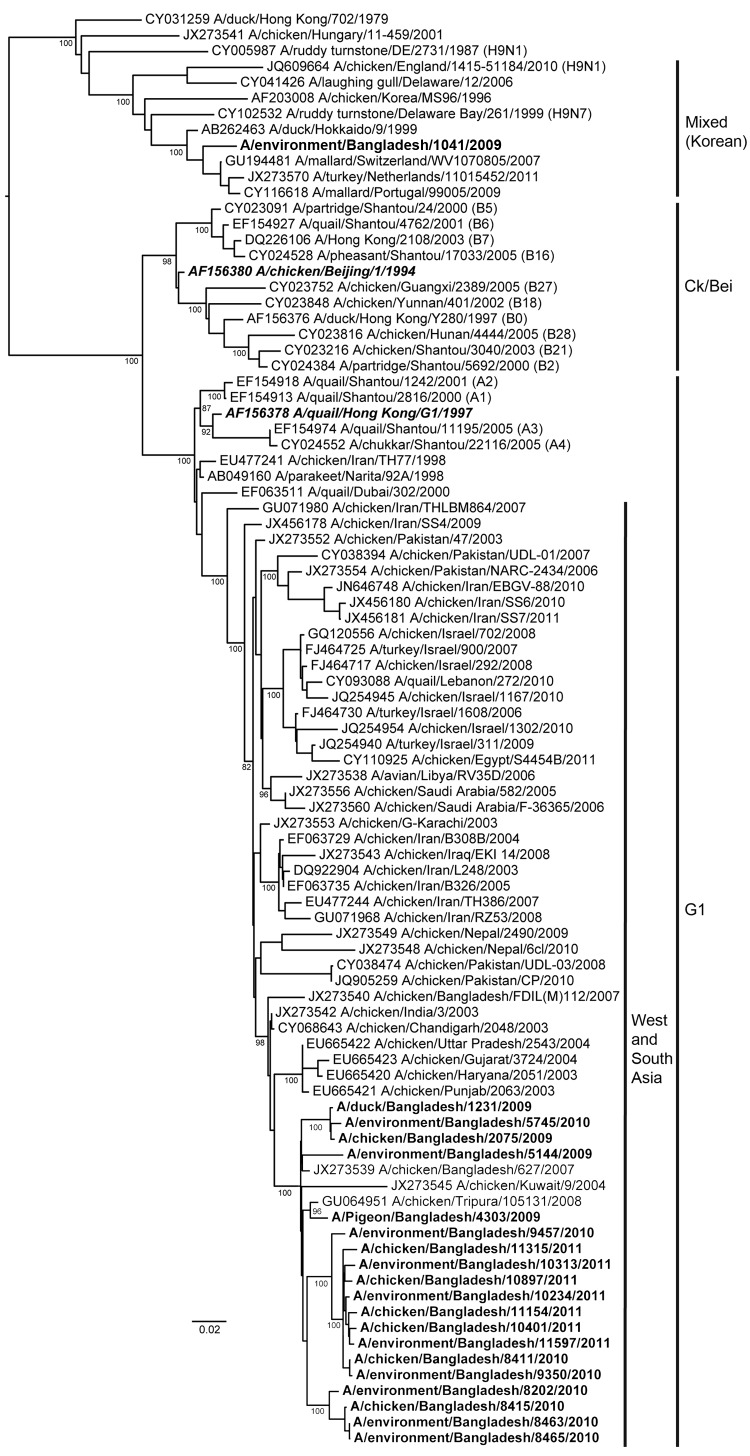
Phylogenetic relationships of hemagglutinin genes of avian influenza (H9N2) viruses (**boldface**) isolated in Bangladesh. Full-length DNA sequencing, starting from the first codon, was used. The phylogenetic trees were generated by PhyML (*30*) within the maximum-likelihood framework. Numbers above the branches indicate bootstrap values; only values >60 are shown. ***Boldface italics*** indicate prototype subtype H9N2 viruses from the Ck/Bei and G1 clades. Scale bar indicates distance between sequence pairs.

Phylogenetically, the HA and NA genes of most of the subtype H9N2 viruses from Bangladesh tightly clustered with those of viruses from India and Pakistan (Figure 2; Technical Appendix Figure). These viruses had >95% sequence homology, grouped within the G1 lineage, and shared a common ancestor with A/Qa/Hk/G1/97. One duck isolate, A/Env/BD/1041/09 (H9N2) (duck), clustered with subtype H9N2 viruses from Korea and shared a common ancestor with A/Dk/HK/Y439/97 (Figure 2). This isolate also showed only 85% nt similarity with the other viruses from Bangladesh.

**Figure 3:**
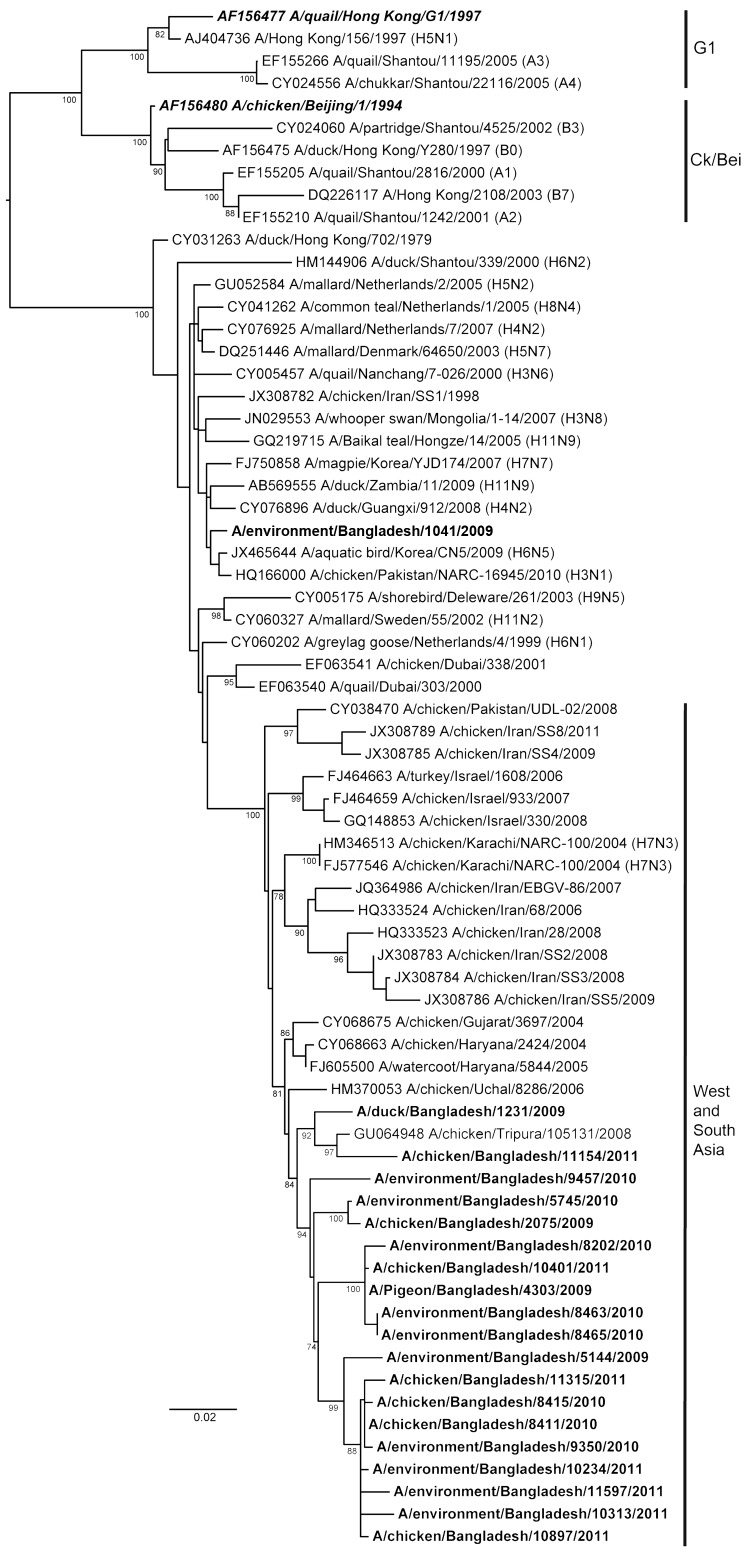
Phylogenetic relationships of nonstructural protein genes of avian influenza (H9N2) viruses (**boldface**) isolated in Bangladesh. Full-length DNA sequencing, starting from the first codon, was used. The phylogenetic trees were generated by PhyML (*30*) within the maximum-likelihood framework. Numbers above the branches indicate bootstrap values; only values >60 are shown. ***Boldface italics*** indicate prototype subtype H9N2 viruses from the Ck/Bei and G1 clades. Scale bar indicates distance between sequence pairs.

Three internal genes (NP, M, and PB2) of the subtype H9N2 viruses from Bangladesh showed a high level of nucleotide homology with the G1-like lineage. All 3 genes were phylogenetically closely related to viruses from India, Pakistan, or the Middle East (Technical Appendix Figure). The duck isolate A/Env/BD/1041/09 (H9N2) (duck) showed similar characteristics in the HA and NA trees, where it shared a common ancestor with a lineage from Korea. The other 3 internal genes (NS, PB1, and PA) of all the subtype H9N2 viruses from Bangladesh grouped into a single cluster, sharing high sequence homology with A/Ck/Karachi/NARC-100/04 (H7N3) (Figure 3; Technical Appendix Figure). These genes formed a distinct clade adjacent to the G1 and Ck/Bei lineages. Although subtype H5N1 viruses were isolated separately and in a mixture with subtype H9N2 viruses, phylogenetic analysis showed no reassortment between the isolates characterized in this study and the subtype H5N1 strains.

### Molecular Characteristics

**Figure 4:**
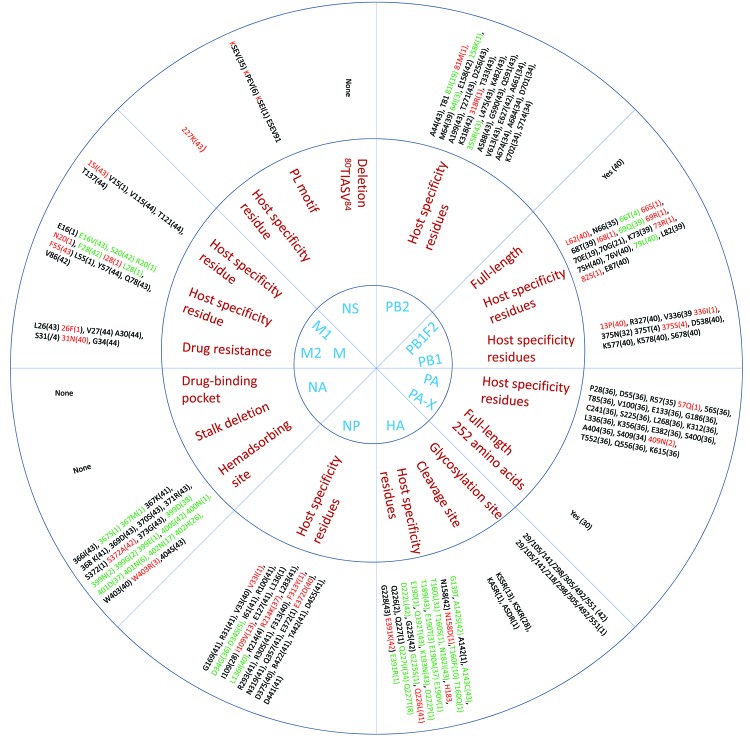
Host range and pathogenicity determinants in avian influenza (H9N2) viruses isolated from different poultry species in Bangladesh during 2008–2011. Numbers in parentheses indicate number of viruses containing specific amino acid residues of the 44 virus isolates analyzed. Blue indicates the 11 genes that were assessed; red indicates the residues that are critical for influenza pathogenesis, enhanced replication in mammalian hosts, or those that are identical to residues present in human influenza viruses; green indicates unique substitutions in the viruses. HA, hemagglutinin; NA, neuraminidase; M, matrix; NS, nonstructural; NP, nucleoprotein; PA and PB, polymerase genes.

To identify the possible determinants of transmission of AIV (H9N2) from birds to humans, we aligned the amino acid sequences of all genes of the subtype H9N2 viruses from Bangladesh and compared them with those of representative subtype H9N2 viruses from different clades (Technical Appendix Tables 2, 3). When compared with the prototype G1 virus, the viruses from Bangladesh showed that they have evolved to acquire mammalian host–specific mutations throughout the genome (Technical Appendix Tables 2, 3). Comparing the amino acid sequences of all genes of the subtype H9N2 viruses from Bangladesh with the prototype subtype H9N2 viruses showed that certain amino acid substitutions throughout the viral genome have become fixed as the viruses have evolved (Figure 4). The receptor-binding site (RBS) of the virus HA influences the generation of human viruses from avian precursors (*1*). Within the RBS, 42 (95.5%) of 44 isolates had leucine (L) at position 226 (H3 numbering), whereas the other 2 viruses had glutamine (Q) at the same position. Of the 2 isolates with 226Q, 1 was a duck virus isolated in early 2009 and 1 was a quail virus isolated in 2011. All viruses with 226L were isolated during 2009–2011 and were found in all of the poultry species sampled. Amino acids at positions 183, 189, 190, and 226 (H3 numbering) are located within the RBS of the HA protein, and the combination of these 4 residues is essential for respiratory droplet transmission of a subtype H9N2 or H3N2 reassortants in ferrets (*6*). Of the 44 viruses analyzed, 42 had 2 (183H and 226L) of the 4 (183H, 189H, 190E, 226L) substitutions. Variations in the number of potential glycosylation sites within the HA are thought to be associated with the adaptation of duck viruses to poultry (*7*). Seven potential glycosylation sites (29, 105,141, 298, 305, 492, and 551) were found in the HAs of 43 (97.7%) of the 44 isolates; the N-X-T/S motif (in which X may be any amino acid except proline) and 2 potential glycosylation sites were lost at positions 208 and 218 (Technical Appendix Tables 2, 3). In the NA proteins of all of the analyzed viruses, we found no R292K substitution, which is associated with resistance to the neuraminidase inhibitor oseltamivir.

Sequence analysis of the internal gene products also identified mammalian host–specific markers (*11*,*14*) that were fixed in the M protein (M1 V15I, 43 isolates; M2 L55F, 44 isolates) and NP (*11*,*14*) (R214K, 37 isolates; E372D, 40 isolates; PB1-L13P, 40 isolates) (Figure 4). In the NS protein, all isolates were full length and possessed the PDZ-(PL) C-terminal motif (*11*,*14*). The NS1 protein also harbored the mammalian-specific glutamate (E) to lysine (K) substitution at position 227 in 43 of the 44 isolates tested (Figure 4).

Our comparison of the deduced amino acid sequences of the subtype H9N2 viruses from Bangladesh revealed that, in positions that were previously identified as important for host specificity, unique substitutions whose functions are unknown have become fixed. In the RBS of the HA protein, the Q227I substitution was in 35 of 44 isolates. This substitution was previously identified in subtype H9N2 viruses from Pakistan and the Middle East but was not found in any of the reference strains. The other unique substitutions were in the M2 protein (E16V and I28F, 43 isolates) and the PB1-F2 protein (R79L, 40 isolates) (Figure 4). Alignment of the M2 proteins showed that 41 of the 44 isolates had a substitution at position 31 (S31N), suggesting resistance to M2-blocker antiviral drugs such as amantadine.

The amino acid sequence of the HA1/HA2-connecting peptide of HA is a major determinant of pathogenicity in terrestrial poultry, and the pathogenicity of highly pathogenic subtype H5N1 and H7N3 viruses is influenced by the presence of a polybasic cleavage site in the connecting peptide (*32*). The amino acid sequences of the cleavage site of the HA1/HA2 junction in the subtype H9N2 viruses from Bangladesh possessed 4 cleavage motifs (Figure 4). Of the 44 isolates we tested, 28 had the KSKR/GLF motif (identified in all recent poultry isolates and a 2011 quail isolate); 14 isolates had the KSSR/GLF motif (identified in chickens, ducks, and older quail isolates); and 1 duck and 1 pigeon isolate had the ASDR/GLF and KASR/GLF motifs, respectively. Thus, the altered amino acid sequences in the connecting peptide of viruses we analyzed were distinct from the RSSR/GLF motif found in the connecting peptides of prototype subtype H9N2 viruses. This finding is clear evidence of genetic variation within isolates from different poultry species.

## Discussion

We isolated a large number of low pathogenicity avian influenza A(H9N2) viruses and an increasing number of highly pathogenic avian influenza A(H5N1) viruses from apparently healthy birds in Bangladesh. During 2008–2011, subtype H9N2 viruses were isolated from multiple poultry species (predominantly chickens and quail) throughout the year, without seasonal prevalence; subtype H5N1 viruses were predominantly isolated from quail during the winter of 2011. Phylogenetic analyses revealed that the subtype H9N2 viruses are of the G1 clade; antigenically, the G1 clade consists of 2 branches distinguished by host species, but the Bangladesh viruses were divergent from the prototype G1 virus. Our comparison of the deduced amino acid sequence showed that the subtype H9N2 viruses have acquired mammalian host–specific mutations in their surface glycoproteins and internal genes.

We isolated subtype H9N2 and H5N1 viruses individually and in viral mixtures; the latter may result from co-infection of the same host species with multiple influenza subtypes. In our surveillance, we isolated influenza subtypes other these than mostly during 2008–2009, with very few other subtypes isolated after 2009. This finding suggests that subtypes H9N2 and H5N1 predominate in live-bird markets.

Phylogenetic analyses showed that, with 1 exception, the isolates we analyzed were homogenous and shared the common ancestor A/Qa/HK/G1/97 (H9N2). G1 was the only lineage identified. The viruses we analyzed shared close nucleotide homology with those of G1 lineage isolated from birds in Pakistan or India, which suggests that the Bangladesh viruses may have their origin in those countries (or vice versa). We speculate that, during the early 2000s, subtype H9N2 viruses circulating in Pakistan were introduced into Bangladesh through regional poultry or pet trade and then established themselves in domestic poultry. Although subtype H9N2 and H5N1 viruses were co-isolated, phylogenetic analysis did not identify any reassortment between the subtypes. However, the subtype H9N2 viruses still maintained the internal genes that were part of the subtype H5N1 reassortant previously implicated in human infection (*7*).

Results from our analyses support our hypothesis that the subtype H9N2 isolates from Bangladesh are related to those from Pakistan and have evolved into 2 distinct subpopulations based on host species. The chicken viruses we analyzed were antigenically homogenous and are related to human viruses but distinct from quail viruses. Furthermore, small changes in the quail viruses should be monitored closely because quail were the host species implicated during the emergence of the subtype H9N2 virus that jumped to humans in 1999 (*7*). In addition, quail were suggested to be the contributing host species of the H9N2/H5N1 reassortant of the subtype H5N1 virus that infected humans in 1997 (*4*)

Our analyses also shows that subtype H9N2 viruses circulating in Bangladesh show a trend toward accumulating molecular markers that favor interspecies transmission. Distinct mutations throughout the viral genomes have been implicated in the adaptation of viruses to a mammalian host (Table 4, Appendix; and Table 5, Appendix). In almost all of the Bangladesh viruses, the RBS of HA has the Q226L substitution, which has been implicated in human virus–like receptor specificity and is critical for replication and direct transmission of these viruses in ferrets (*33**,**34*).

**Table 4:** HA, NA, and M gene substitutions that favor replication in mammals in avian influenza A(H9N2) viruses isolated from poultry in Bangladesh during 2008–2011*†

Residues and isolates	HA									NA			M1		M2 drug resistance					
HA RBS					HA1/HA2		Glyco site	HB site		15	16	20	28	55	26	31
Residues																				
H9N2 numbering	166	191	234	399				218		372	403									
H3 numbering	158	183	226	391																
Isolates																				
A/Env/BD/907/2009	N	**H**	**L**	**K**		KSSR		No		**A**	W		**I**		V	S	F	**F**	L	**N**
A/Env/BD1041/2009	**S**	**H**	Q	**K**		ASDR		**NRTF**		S	W		V		E	S	**I**	L	L	S
A/Ck/BD/2075/2009	N	**H**	**L**	**K**		KSSR		No		**A**	W		**I**		V	S	F	**F**	L	S
A/Env/BD/8202/2010	N	**H**	**L**	**K**		KSSR		No		**A**	W		**I**		V	**N**	F	**F**	**F**	**N**
A/Ck/BD/8996/2010	N	**H**	**L**	**K**		KSKR		No		**A**	W		**I**		V	S	F	**F**	L	**N**
A/Ck/BD/9334/2010	N	**H**	**L**	**K**		KSKR		No		**A**	W		**I**		V	S	F	**F**	L	**N**
A/Env/BD/10234/2011	N	**H**	**L**	**K**		KSKR		No		**A**	W		**I**		V	S	F	**F**	L	**N**
A/Env/BD/10306/2011	**D**	**H**	Q	**K**		KSKR		No		**A**	W		**I**		V	S	F	**F**	L	**N**
A/Ck/BD/10450/2011	N	**H**	**L**	**K**		KSKR		No		**A**	W		**I**		V	S	F	**F**	L	**N**
A/Ck/BD/10897/2011	N	**H**	**L**	**K**		KSKR		No		**A**	W		**I**		V	S	F	**F**	L	**N**
A/Ck/BD/11154/2011	N	**H**	**L**	**K**		KSKR		No		**A**	W		**I**		V	S	F	**F**	L	**N**
A/Env/BD/11173/2011	N	**H**	**L**	**K**		KSKR		No		**A**	W		**I**		V	S	F	**F**	L	**N**
A/Env/BD/11309/2011	N	**H**	**L**	**K**		KSKR		No		**A**	W		**I**		V	S	F	**F**	L	**N**
A/Env/BD/11315/2011	N	**H**	**L**	**K**		KSKR		No		**A**	W		**I**		V	S	F	**F**	L	**N**
A/Env/BD/12116/2011	N	**H**	**L**	**K**		KSKR		No		ND	ND		**I**		V	S	F	**F**	L	**N**
A/Env/BD/12119/2011	N	**H**	**L**	**K**		KSKR		No		**A**	W		**I**		V	S	F	**F**	L	**N**
A/Env/BD/13916/2011	N	**H**	**L**	**K**		KSSR		No		**A**	**R**		**I**		V	S	F	**F**	L	**N**
A/Ck/BD/13962/2011	N	**H**	**L**	**K**		KSKR		No		**A**	W		**I**		V	S	F	**F**	L	**N**

*A total of 31 unique substitutions were identified in 18 H9 isolates. Isolates 8202 (M2 adamantine-like drug resistance mutation), 10306 (HA158D) and13916 (NA403R) contain unique substitution not seen in most isolates. **Boldface** indicates key amino acid substitutions implicated in mammalian transmission. HA, hemagglutinin; NA, neuraminidase; M, matrix; RBS, receptor-binding site; glyco, glycosylation; HB, hemabsorbing; ND, analysis not done because sequences were not available. †Amino acids: A, alanine; D, aspartic acid; E, glutamic acid; H, histidine; I, isoleucine; K, lysine; L, leucine; N, asparagine; Q, glutamine; R, arginine; S, serine; V, valine.

**Table 5:** NS, NP, PA, PB1, and PB2 gene substitutions that favor replication in mammals in avian influenza A(H9N2) viruses isolated from poultry in Bangladesh during 2008–2011*†

Isolate	NS			NP						PA		PA-X	PB1				PB1-F2					PB2	
	NS1 (227)	PL motif		33	109	214	313	372		57	409		13	336	375		66	68	73	82		81	318
A/Env/BD/907/2009	**K**	**K**SEV		V	I	**K**	F	**D**		R	S	Yes	**P**	V	N		N	T	K	**S**		T	K
A/Env/BD1041/2009	E	ESEV		V	I	R	F	E		R	S		ND	ND	ND		ND	ND	ND	ND		T	**R**
A/Ck/BD/2075/2009	**K**	**K**SEV		V	I	R	F	**D**		R	S		**P**	V	T		N	T	K	L		T	K
A/Env/BD/8202/2010	**K**	**K**PEV		V	**V**	**K**	**Y**	**D**		R	S		**P**	V	**S**		N	T	K	L		**I**	K
A/Ck/BD/8996/2010	**K**	**K**SEV		V	**V**	**K**	F	**D**		R	**N**		**P**	V	**S**		N	T	K	L		I	K
A/Ck/BD/9334/2010	ND	ND		**I**	I	**K**	F	**D**		R	S		**P**	V	N		N	T	K	L		T	K
A/Env/BD/10234/2011	**K**	**K**SEV		V	**V**	**K**	F	**D**		R	S		**P**	V	**S**		N	T	K	L		I	K
A/Env/BD/10306/2011	**K**	**K**SEV		V	**V**	**K**	F	**D**		R	S		**P**	V	N		N	**I**	K	L		I	K
A/Ck/BD/10450/2011	**K**	**K**SEV		V	**V**	**K**	F	**D**		R	S		**P**	V	N		N	T	K	L		T	K
A/Ck/BD/10897/2011	**K**	**K**SEV		V	I	**K**	F	**D**		R	S		**P**	V	**S**		N	T	K	L		T	K
A/Ck/BD/11154/2011	**K**	**K**SEV		V	**V**	**K**	F	**D**		R	S		**P**	V	N		N	T	K	L		T	K
A/Env/BD/11173/2011	**K**	**K**SEV		V	**V**	**K**	F	**D**		R	S		**P**	V	N		N	T	K	L		I	K
A/Env/BD/11309/2011	**K**	**K**SEV		V	**V**	**K**	F	**D**		R	S		**P**	V	N		N	T	K	L		ND	ND
A/Env/BD/11315/2011	**K**	**K**SEV		V	**V**	**K**	F	**D**		R	S		**P**	V	N		N	T	K	L		I	K
A/Env/BD/12116/2011	**K**	**K**SEV		V	**V**	**K**	F	**D**		ND	ND		**P**	V	N		N	T	K	L		I	K
A/Env/BD/12119/2011	**K**	**K**SEV		V	**V**	**K**	F	**D**		ND	ND		**P**	V	N		N	T	K	L		I	K
A/Env/BD/13916/2011	**K**	**K**SEV		V	I	**K**	F	**D**		ND	ND		**P**	**I**	N		N	T	K	L		T	K
A/Ck/BD/13962/2011	**K**	**K**SEV		V	**V**	**K**	F	**D**		ND	ND		**P**	V	N		**S**	T	**R**	L		I	K

*A total of 31 unique substitutions were identified in 18 H9 isolates. Isolates 907 (PB1-F2, 82S), 8202 (NP-313Y), 10306 (PB1-F2 68I), 10897 (PB1375S), 13916 (PB1336I), and 13962(PB1-F2 66S/82R) contain unique substitution not seen in most isolates. **Boldface** indicates key amino acid substitutions implicated in mammalian transmission. NS, nonstructural; NP, nucleoprotein; PA and PB, polymerase genes; ND, analysis not done because sequences were not available. †Amino acids: D, aspartic acid; E, glutamic acid; I, isoleucine; K, lysine; L, leucine; N, asparagine; R, arginine; S, serine; T, threonine; V, valine.

Although the viruses we isolated have low pathogenicity and do not possess a polybasic cleavage site, they carry a motif similar to cleavage sites seen in the highly pathogenic H5N1 and H7N3 viruses. This result suggests that subtype H9N2 viruses have the potential to acquire a polybasic site and become highly pathogenic. In the HA of nearly all the isolates that we analyzed, 2 potential glycosylation sites were lost; this process was also seen in the adaptation of duck viruses to poultry (*32**,**35*). Because the duck isolate A/Env/BD/1041/2009 retained its glycosylation site, we hypothesize that the introduction of the Y439 lineage via ducks was unsuccessful because the G1 lineage was already dominant in the local poultry species.

In most of the viruses we analyzed, we observed residues in the avian–human signature positions becoming fixed (HA 226L and 183H, M1 V15I, M2 L55F, NS E227K, NP R214K, E372D, PB1-L13P); in certain isolates, the mutations were fixed by unique substitutions whose functions are unknown (HA Q227I, M2 E16V, I28F, PB1-F2 R79L). In addition, these viruses have acquired substitutions similar to those seen in highly pathogenic subtype H5N1 and H7N3 and pandemic subtype H1N1 and H3N2 viruses (36). Noticeably, in the NS gene’s PDZ motif, most subtype H9N2 isolates carried the KSEV motif seen in the 1918 pandemic subtype H1N1 virus (*11*,*37*). In the PA genes of 2 subtype H9N2 viruses, we identified R57Q and S409N substitutions, both of which were in the 1968 pandemic subtype H3N2 virus. These observations confirm that the Bangladesh subtype H9N2 viruses have accumulated molecular markers that influence host specificity and pathogenesis.

In conclusion, our results show that the Bangladesh subtype H9N2 viruses are genetically similar to, but distinct from, the A/Qa/HK/G1/97 isolate, which has been previously implicated in human infection (*7*). However, the Bangladesh viruses have accumulated molecular characteristics required for infecting humans and are antigenically similar to the human subtype H9N2 virus isolated from a patient in Bangladesh. These viruses have a tendency to reassort with highly pathogenic subtype H5N1 (*7*) and H7 viruses (*38**,**39*), which is particularly concerning in light of the emergence of avian influenza A(H7N9) virus in China that contains 6 gene segments from H9N2 influenza viruses and causes lethal infection in humans (*40*). The emergence of this reassortant virus emphasizes the potential for, and the danger of, transmission of subtype H9N2 viruses to humans.

Technical Appendix Genes of influenza A(H9N2) viruses from Bangladesh that were not sequenced or only partially sequenced, amino acid substitutions in hemagglutinin and neuraminidase gene products and in internal genes that determine host specificity of the viruses, and phylogenetic relations of key internal genes of the virus isolates.
