# Supplementary material for: Antigenic and Molecular Characterization of Avian Influenza A(H9N2) Viruses, Bangladesh
Source: Emerg Infect Dis. 2013 Sep;19(9):1393–402. doi: 10.3201/eid1909.130336 (PMC3810925; doi:10.3201/eid1909.130336)
Supplement: Technical Appendix — Genes of influenza A(H9N2) viruses from Bangladesh that were not sequenced or only partially sequenced, amino acid substitutions in hemagglutinin and neuraminidase gene products and in internal genes that determine host specificity of the viruses, and phylogenetic relations of key internal genes of the virus isolates. [file 13-0336-Techapp-s1.pdf]

Article DOI: http://dx.doi.org/10.3201/eid1909.130336

Antigenic and Molecular Characterization of Avian Influenza A(H9N2) Viruses, Bangladesh

Technical Appendix

Technical Appendix Table 1. Genes of influenza A(H9N2) viruses from Bangladesh that were either not sequenced (X) or only partially sequenced (P)

	Influenza virus gene						
Virus isolate	NA	NP	NS	PA	PB1	PB2	HA
A/Environment/Bangladesh/907/2009							
A/Environment/Bangladesh/1041/2009					X		
A/Duck/Bangladesh/1231/2009							
A/Chicken/Bangladesh/2075/2009							
A/Pigeon/Bangladesh/4303/2009							
A/Environment/Bangladesh/5144/2009							
A/Chicken/Bangladesh/5209/2009							
A/Environment/Bangladesh/5745/2010							
A/Environment/Bangladesh/8202/2010							
A/Chicken/Bangladesh/8411/2010						P	
A/Chicken/Bangladesh/8413/2010							
A/Chicken/Bangladesh/8415/2010						P	
A/Environment/Bangladesh/8463/2010							
A/Environment/Bangladesh/8465/2010						P	
A/Chicken/Bangladesh/8725/2010					X		
A/Chicken/Bangladesh/8731/2010				X		P	
A/Chicken/Bangladesh/8996/2010						P	
A/Chicken/Bangladesh/9029/2010						P	
A/Environment/Bangladesh/9306/2010				X			
A/Chicken/Bangladesh/9334/2010			X				
A/Environment/Bangladesh/9350/2010						P	
A/Environment/Bangladesh/9457/2010						P	
A/Environment/Bangladesh/10234/2011							
A/Environment/Bangladesh/10306/2011							
A/Environment/Bangladesh/10307/2011						P	
A/Environment/Bangladesh/10313/2011							
A/Environment/Bangladesh/10316/2011			X				
A/Chicken/Bangladesh/10401/2011							
A/Chicken/Bangladesh/10411/2011							X
A/Chicken/Bangladesh/10450/2011						P	
A/Chicken/Bangladesh/10897/2011							
A/Chicken/Bangladesh/11154/2011							
A/Environment/Bangladesh/11173/2011							
A/Chicken/Bangladesh/11309/2011						X	
A/Chicken/Bangladesh/11315/2011							
A/Environment/Bangladesh/11597/2011							
A/Environment/Bangladesh/12068/2011		X					
A/Environment/Bangladesh/12077/2011							
A/Environment/Bangladesh/12093/2011		X			X		
A/Environment/Bangladesh/12103/2011		X		X			
A/Environment/Bangladesh/12116/2011	X			X			
A/Environment/Bangladesh/12119/2011				X			
A/Chicken/Bangladesh/13916/2011				X			
A/Chicken/Bangladesh/13962/2011				X			

Technical Appendix Table 2. Amino acid substitutions* in hemagglutinin and neuraminidase gene products that determine host specificity of influenza A(H9N2) viruses from Bangladesh

	HA											NA							
	HA RBS									HA1/HA2 Cleavage site	Glycosylation site		Stalk Deletion			Hemabsorbing site			
Residues at HA RBS (H9N2 numbering)	166	191	197	198	232	234	235	236	399		206	218	38-39	46-50	62-64	372	402	403	431-433
H3 Residues at HA RBS (H3 numbering)	158	183	189	190	224	226	227	228	391										
A/Qa/HK/G1/97	S	H	T	E	N	L	Q	G	K	RSSR	NDTT	NRTF	Yes	No	No	S	I	R	PQE
A/Ck/HK/G9/97	N	N	T	A	N	L	Q	G	K	RSSR	No	NRTF	No	No	No	S	N	S	PKE
A/Dk/HK/Y280/97	N	N	T	T	N	L	Q	G	K	RSSR	No	NRTF	No	No	No	S	N	W	PQE
A/Ck/Bei/1/94	N	N	T	V	N	Q	Q	G	K	RSSR	No	NRTF	No	No	No	S	N	W	PQE
A/Dk/HK/Y439/97	S	H	T	E	N	Q	Q	G	K	ASYR	No	NRTF	No	No	No	S	N	W	PQE
A/HK/1073/99	S	H	T	E	N	L	Q	G	K	RSSR	NDTT	NRTF	Yes	No	No	S	N	W	PQE
A/Env/BD/907/2009	N	**H**	T	T	N	**L**	I	G	**K**	KSSR	No	No		No		**A**	N	W	PQE
A/Env/BD/1041/2009	**S**	**H**	T	**E**	N	Q	Q	G	**K**	ASDR	No	**NRTF**				S	N	W	PQE
A/Dk/BD/1231/2009	N	**H**	T	A	N	**L**	I	G	**K**	KSSR	No	No				**A**	N	W	PQE
A/Ck/BD/2075/2009	N	**H**	T	A	N	**L**	I	G	**K**	KSSR	No	No				**A**	N	W	PQE
A/Pg/BD/4303/2009	N	**H**	T	A	N	**L**	I	G	**K**	KASR	No	No				**A**	N	W	PQE
A/Env/BD/5144/2009	N	**H**	T	T	N	**L**	I	G	**K**	KSSR	No	No				**A**	N	W	PQE
A/Ck/BD/5209/2009	N	**H**	T	A	N	**L**	I	G	**K**	KSSR	No	No				**A**	N	W	PQE
A/Env/BD/5745/2010	N	**H**	T	A	N	**L**	I	G	**K**	KSSR	No	No				**A**	N	W	PQE
A/Env/BD/8202/2010	N	**H**	T	A	N	**L**	I	G	**K**	KSSR	No	No				**A**	N	W	PQE
A/Ck/BD/8411/2010	N	**H**	T	A	N	**L**	I	G	**K**	KSKR	No	No				**A**	H	W	PQE
A/Ck/BD/8413/2010	N	**H**	T	A	N	**L**	I	G	**K**	KSSR	No	No				**A**	H	W	PQE
A/Ck/BD/8415/2010	N	**H**	T	A	N	**L**	I	G	**K**	KSSR	No	No				**A**	H	W	PQE
A/Env/BD/8463/2010	N	**H**	T	A	N	**L**	I	G	**K**	KSSR	No	No				**A**	N	W	PQE
A/Env/BD/8465/2010	N	**H**	T	A	N	**L**	I	G	**K**	KSSR	No	No				**A**	N	W	PQE
A/Ck/BD/8725/2010	N	**H**	T	A	N	**L**	I	G	**K**	KSKR	No	No				**A**	H	W	PQE
A/Ck/BD/8731/2010	N	**H**	T	A	N	**L**	I	G	**K**	KSKR	No	No				**A**	H	W	PQE
A/Ck/BD/8996/2010	N	**H**	T	A	N	**L**	I	G	**K**	KSKR	No	No				**A**	H	W	PQE
A/Ck/BD/9029/2010	N	**H**	T	A	N	**L**	I	G	**K**	KSSR	No	No				**A**	N	W	PQE
A/Env/BD/9306/2010	N	**H**	T	A	N	**L**	I	G	**K**	KSKR	No	No				**A**	H	W	PQE
A/Ck/BD/9334/2010	N	**H**	T	A	N	**L**	I	G	**K**	KSKR	No	No				**A**	H	W	PQE
A/Env/BD/9350/2010	N	**H**	T	A	N	**L**	I	G	**K**	KSKR	No	No				**A**	H	W	PQE
A/Env/BD/9457/2010	N	**H**	T	A	N	**L**	I	G	**K**	KSSR	No	No				**A**	H	W	PQE
A/Env/BD/10234/2011	N	**H**	T	A	N	**L**	I	G	**K**	KSKR	No	No				**A**	N	W	PQE
A/Env/BD/10306/2011	**D**	**H**	T	A	N	Q	T	G	**K**	KSKR	No	No				**A**	H	W	PQE
A/Env/BD/10307/2011	N	**H**	T	T	N	**L**	T	G	**K**	KSKR	No	No				**A**	H	W	PQE
A/Env/BD/10313/2011	N	**H**	T	A	N	**L**	T	G	**K**	KSKR	No	No				**A**	H	W	PQE
A/Env/BD/10316/2011	N	**H**	T	A	N	**L**	T	G	**K**	KSKR	No	No				**A**	H	W	PQE
A/Ck/BD/10401/2011	N	**H**	T	A	N	**L**	I	G	**K**	KSKR	No	No				**A**	N	W	PQE
A/Ck/BD/10411/2011	N	**H**	T	A	N	**L**	I	G	**K**	KSKR	No	No				**A**	H	W	PQE
A/Ck/BD/10450/2011	N	**H**	T	A	N	**L**	I	G	**K**	KSKR	No	No				**A**	H	W	PQE
A/Ck/BD/10897/2011	N	**H**	T	A	N	**L**	I	G	**K**	KSKR	No	No				**A**	H	W	PQE
A/Ck/BD/11154/2011	N	**H**	T	A	N	**L**	I	G	**K**	KSKR	No	No				**A**	H	W	PQE
A/Env/BD/11173/2011	N	**H**	T	A	N	**L**	I	G	**K**	KSKR	No	No				**A**	H	W	PQE
A/Ck/BD/11309/2011	N	**H**	T	A	N	**L**	I	G	**K**	KSKR	No	No				**A**	H	W	PQE

Publisher: CDC; Journal: Emerging Infectious Diseases
Article Type: Research; Volume: 19; Issue: 9; Year: 2013; Article ID: 13-0336
DOI: 10.3201/eid1909.130336; TOC Head: Research

	HA												NA					
	HA RBS									HA1/HA2 Cleavage site	Glycosylation site		Stalk Deletion	Hemabsorbing site			
A/Ck/BD/11315/2011	N	**H**	T	A	N	**L**	I	G	**K**	KSKR	No	No		**A**	H	W	PQE
A/Env/BD/11597/2011	N	**H**	T	A	N	**L**	I	G	R	KSKR	No	No		**A**	N	**R**	PQE
A/Env/BD/12068/2011	N	**H**	T	I	N	**L**	I	G	**K**	KSKR	No	No		**A**	H	W	PQE
A/Env/BD/12077/2011	N	**H**	T	A	N	**L**	I	G	**K**	KSKR	No	No		**A**	N	**R**	PQE
A/Env/BD/12093/2011	N	**H**	T	A	N	**L**	T	G	**K**	KSKR	No	No		**A**	H	W	PQE
A/Env/BD/12103/2011	N	**H**	T	V	N	**L**	T	G	**K**	KSKR	No	No		**A**	H	W	PQE
A/Env/BD/12116/2011	N	**H**	T	A	N	**L**	T	G	**K**	KSKR	No	No		na	na	na	na
A/Env/BD/12119/2011	N	**H**	T	A	N	**L**	T	G	**K**	KSKR	No	No		**A**	H	W	PQE
A/Ck/BD/13916/2011	N	**H**	T	A	N	**L**	I	G	**K**	KSSR	No	No		**A**	N	**R**	PQE
A/Ck/BD/13962/2011	N	**H**	T	A	N	**L**	I	G	**K**	KSKR	No	No		**A**	H	W	PQE

*Key amino acid substitutions are formatted in **boldface**; na, sequence not available for analyses.
Abbreviations: A, alanine; D, aspartic acid; E, glutamic acid; G, glycine; H, histidine; I, isoleucine; K, lysine; L, leucine; N, asparagine; Q, glutamine; R, arginine; S, serine; T, threonine; V, valine.

Technical Appendix Table 3. Amino acid substitutions* in the internal genes that influence the host specificity of influenza A(H9N2) viruses from Bangladesh

	M1	M2				M2 drug resistance		NS			NP					PA		PB1			PB1-F2						PB2	
	15	16	20	28	55	26	31	Deletion of amino acid ^{80}TIASV^{84}	NS1 (227)	PL motif	33	109	214	313	372	57	409	13	336	375	Size (aa)	66	68	73	79	82	81	318
Predicted Avian	V	E	S	I	L				E		V	I	R	F	E	R	T	L	V					K	R	L	T	
Predicted Mammalian	I	G/D	N	I/V	F				R/K		I	V	K	Y	D	Q	S	P	I					R	Q	S	M	
Predicted amantadine resistant						F	N/G																					
Predicted amantadine sensitive						L	S																					
A/Qa/HK/G1/97	I	G	S	V	F	L	S	No	E	EPEV	V	I	R	F	E	R	S	P	V	N							T	K
A/Ck/HK/G9/97	I	G	S	V	F	L	S		E	EPEV	V	I	R	F	E	R	S	P	V	N							I	R
A/Dk/HK/Y280/97	I	G	S	V	F	L	S		E	EPEV	V	I	R	F	E	R	S	P	V	T							T	R
A/Ck/Bei/1/94	I								E	EPEV				F	E	R	S	P	V								-	-
A/Dk/HK/Y439/97	V								E	ESEV	V	I	R	F	E	R	S	P	V	N							T	R
A/HK/1073/99	I								E	EPEV	V	I	R	F	E	R	S	P	V	N							T	R
A/Env/BD/907/2009	**I**	V	S	F	**F**	L	**N**	No	**K**	**K**SEV	V	I	**K**	F	**D**	R	S	**P**	V	N	90	N	T	K	L	S	T	K
A/Env/BD/1041/2009	V	E	S	**I**	L	L	S		E	ESEV	V	I	R	F	E	R	S	na	na	na	na	na	na	na	na	na	T	**R**
A/Dk/BD/1231/2009	**I**	V	S	F	**F**	L	**N**		**K**	**K**SEV	V	I	**K**	F	**D**	R	S	**P**	V	N	90	N	T	K	L	L	T	K
A/Ck/BD/2075/2009	**I**	V	**N**	F	**F**	L	S		**K**	**K**SEV	V	I	R	F	**D**	R	S	**P**	V	T	90	N	T	K	L	L	T	K
A/Pg/BD/4303/2009	**I**	V	S	F	**F**	L	**N**		**K**	**K**PEV	V	I	**K**	F	**D**	R	S	**P**	V	N	90	N	T	K	L	L	I	K
A/Env/BD/5144/2009	**I**	V	S	F	**F**	L	S		**K**	**K**SEV	V	I	R	F	**D**	R	S	na	na	na	na	na	na	na	na	na	T	K
A/Ck/BD/5209/2009	**I**	V	S	F	**F**	L	**N**		**K**	**K**SEV	V	I	R	F	**D**	R	S	**P**	V	N	90	N	T	K	L	L	T	K
A/Env/BD/5745/2010	**I**	V	S	F	**F**	L	**N**		**K**	**K**SEV	V	I	**K**	F	**D**	R	S	**P**	V	N	90	N	T	K	L	L	T	K
A/Env/BD/8202/2010	**I**	V	S	F	**F**	**F**	**N**		**K**	**K**PEV	V	**V**	**K**	**Y**	**D**	R	S	**P**	V	**S**	90	N	T	K	L	L	I	K
A/Ck/BD/8411/2010	**I**	V	S	F	**F**	L	**N**		**K**	**K**SEV	V	I	**K**	F	**D**	R	S	**P**	V	N	90	N	T	K	L	L	T	K
A/Ck/BD/8413/2010	**I**	V	S	F	**F**	L	**N**		**K**	**K**SEV	V	I	**K**	F	**D**	R	S	**P**	V	N	90	N	T	K	L	L	T	K
A/Ck/BD/8415/2010	**I**	V	S	F	**F**	L	**N**		**K**	**K**SEV	V	I	**K**	F	**D**	R	S	**P**	V	N	90	N	T	K	L	L	T	K
A/Env/BD/8463/2010	**I**	V	S	F	**F**	L	**N**		**K**	**K**PEV	V	I	**K**	F	**D**	R	S	**P**	V	N	90	N	T	K	L	L	T	K

	M1	M2				M2 drug resistance		NS			NP					PA		PB1			PB1-F2						PB2	
	15	16	20	28	55	26	31	Deletion of amino acid ^{80}TIASV^{84}	NS1 (227)	PL motif	33	109	214	313	372	57	409	13	336	375	Size (aa)	66	68	73	79	82	81	318
A/Env/BD/8465/2010	**I**	V	S	F	**F**	L	**N**		**K**	**K**PEV	V	I	**K**	F	**D**	R	S	**P**	V	N	90	N	T	K	L	L	T	K
A/Ck/BD/8725/2010	**I**	V	S	F	**F**	L	**N**		**K**	**K**SEV	V	I	**K**	F	**D**	R	S	na	na	na	na	na	na	na	na	na	T	K
A/Ck/BD/8731/2010	**I**	V	S	F	**F**	L	**N**		**K**	**K**SEV	V	I	**K**	F	**D**	na	na	**P**	V	N	90	N	T	K	L	L	T	K
A/Ck/BD/8996/2010	**I**	V	S	F	**F**	L	**N**		**K**	**K**SEV	V	**V**	**K**	F	**D**	R	**N**	**P**	V	**S**	90	N	T	K	L	L	I	K
A/Ck/BD/9029/2010	**I**	V	S	F	**F**	L	**N**		**K**	**K**PEV	V	I	**K**	F	**D**	R	S	**P**	V	T	90	N	T	K	L	L	I	K
A/Env/BD/9306/2010	**I**	V	S	F	**F**	L	**N**		**K**	**K**SEV	V	I	**K**	F	**D**	na	na	**P**	V	N	90	N	T	K	L	L	T	K
A/Ck/BD/9334/2010	**I**	V	S	F	**F**	L	**N**		na	na	**I**	I	**K**	F	**D**	R	S	**P**	V	N	90	N	T	K	L	L	T	K
A/Env/BD/9350/2010	**I**	V	S	F	**F**	L	**N**		**K**	**K**SEV	V	I	**K**	F	**D**	R	S	**P**	V	N	90	N	T	K	L	L	T	K
A/Env/BD/9457/2010	**I**	V	S	F	**F**	L	**N**		**K**	**K**SEI	V	**V**	**K**	F	**D**	R	S	**P**	V	N	90	N	T	K	L	L	I	K
A/Env/BD/10234/2011	**I**	V	S	F	**F**	L	**N**		**K**	**K**SEV	V	**V**	**K**	F	**D**	R	S	**P**	V	**S**	90	N	T	K	L	L	I	K
A/Env/BD/10306/2011	**I**	V	S	F	**F**	L	**N**		**K**	**K**SEV	V	**V**	**K**	F	**D**	R	S	**P**	V	N	90	N	**I**	K	L	L	I	K
A/Env/BD/10307/2011	**I**	V	S	F	**F**	L	**N**		**K**	**K**SEV	V	I	**K**	F	**D**	R	S	**P**	V	N	90	N	T	K	L	L	*	K
A/Env/BD/10313/2011	**I**	V	S	F	**F**	L	**N**		**K**	KSEV	V	I	**K**	F	**D**	R	S	**P**	V	N	90	N	T	K	L	L	I	K
A/Env/BD/10316/2011	**I**	V	S	F	**F**	L	**N**		na	na	V	I	**K**	F	**D**	R	S	**P**	V	N	90	N	T	K	L	L	I	K
A/Ck/BD/10401/2011	**I**	V	S	F	**F**	L	**N**		**K**	**K**PEV	V	I	**K**	F	**D**	R	**N**	**P**	V	T	90	N	T	K	L	L	I	K
A/Ck/BD/10411/2011	**I**	V	S	F	**F**	L	**N**		**K**	**K**SEV	V	I	**K**	F	**D**	R	S	**P**	V	N	90	N	T	K	L	L	T	K
A/Ck/BD/10450/2011	**I**	V	S	F	**F**	L	**N**		**K**	**K**SEV	V	**V**	**K**	F	**D**	R	S	**P**	V	N	90	N	T	K	L	L	T	K
A/Ck/BD/10897/2011	**I**	V	S	F	**F**	L	**N**		**K**	**K**SEV	V	I	**K**	F	**D**	R	S	**P**	V	**S**	90	N	T	K	L	L	T	K
A/Ck/BD/11154/2011	**I**	V	S	F	**F**	L	**N**		**K**	**K**SEV	V	**V**	**K**	F	**D**	R	S	**P**	V	N	90	N	T	K	L	L	T	K
A/Env/BD/11173/2011	**I**	V	S	F	**F**	L	**N**		**K**	**K**SEV	V	**V**	**K**	F	**D**	R	S	**P**	V	N	90	N	T	K	L	L	I	K
A/Ck/BD/11309/2011	**I**	V	S	F	**F**	L	**N**		**K**	**K**SEV	V	**V**	**K**	F	**D**	R	S	**P**	V	N	90	N	T	K	L	L	na	na
A/Ck/BD/11315/2011	**I**	V	S	F	**F**	L	**N**		**K**	**K**SEV	V	**V**	**K**	F	**D**	R	S	**P**	V	N	90	N	T	K	L	L	I	K
A/Env/BD/11597/2011	**I**	V	R	F	**F**	L	**N**		**K**	**K**SEV	V	I	**K**	F	**D**	R	S	**P**	V	N	90	N	T	K	L	L	I	K
A/Env/BD/12068/2011	**I**	V	S	F	**F**	L	**N**		**K**	**K**SEV	na	na	na	na	na	R	S	**P**	V	T	90	N	T	K	L	L	I	K
A/Env/BD/12077/2011	**I**	V	S	L	**F**	L	S		**K**	**K**SEV	V	I	K	F	**D**	**Q**	S	**P**	V	N	90	N	T	K	L	L	T	K
A/Env/BD/12093/2011	**I**	V	S	F	**F**	L	**N**		**K**	**K**SEV	na	na	na	na	na	R	S	na	na	na	na	na	na	na	na	na	I	K
A/Env/BD/12103/2011	**I**	V	S	F	**F**	L	**N**		**K**	**K**SEV	na	na	na	na	na	na	na	**P**	V	N	90	N	T	K	L	L	I	K
A/Env/BD/12116/2011	**I**	V	S	F	**F**	L	**N**		**K**	**K**SEV	V	**V**	**K**	F	**D**	na	na	**P**	V	N	90	N	T	K	L	L	I	K
A/Env/BD/12119/2011	**I**	V	S	F	**F**	L	**N**		**K**	**K**SEV	V	**V**	**K**	F	**D**	na	na	**P**	V	N	90	N	T	K	L	L	I	K
A/Ck/BD/13916/2011	**I**	V	S	F	**F**	L	**N**		**K**	**K**SEV	V	I	**K**	F	**D**	na	na	**P**	I	N	90	N	T	K	L	L	T	K
A/Ck/BD/13962/2011	**I**	V	S	F	**F**	L	**N**		**K**	**K**SEV	V	**V**	**K**	F	**D**	na	na	**P**	V	N	90	**S**	T	R	L	L	I	K

*Key amino acid substitutions are formatted in **boldface**; na, sequence not available for analyses.
Abbreviations: D, aspartic acid; E, glutamic acid; F, phenylalanine; G, glycine; I, isoleucine; K, lysine; L, leucine; N, asparagine; M, methionine; P, proline; Q, glutamine; R, arginine; S, serine; T, threonine; V, valine.

Publisher: CDC; Journal: Emerging Infectious Diseases
Article Type: Research; Volume: 19; Issue: 9; Year: 2013; Article ID: 13-0336
DOI: 10.3201/eid1909.130336; TOC Head: Research

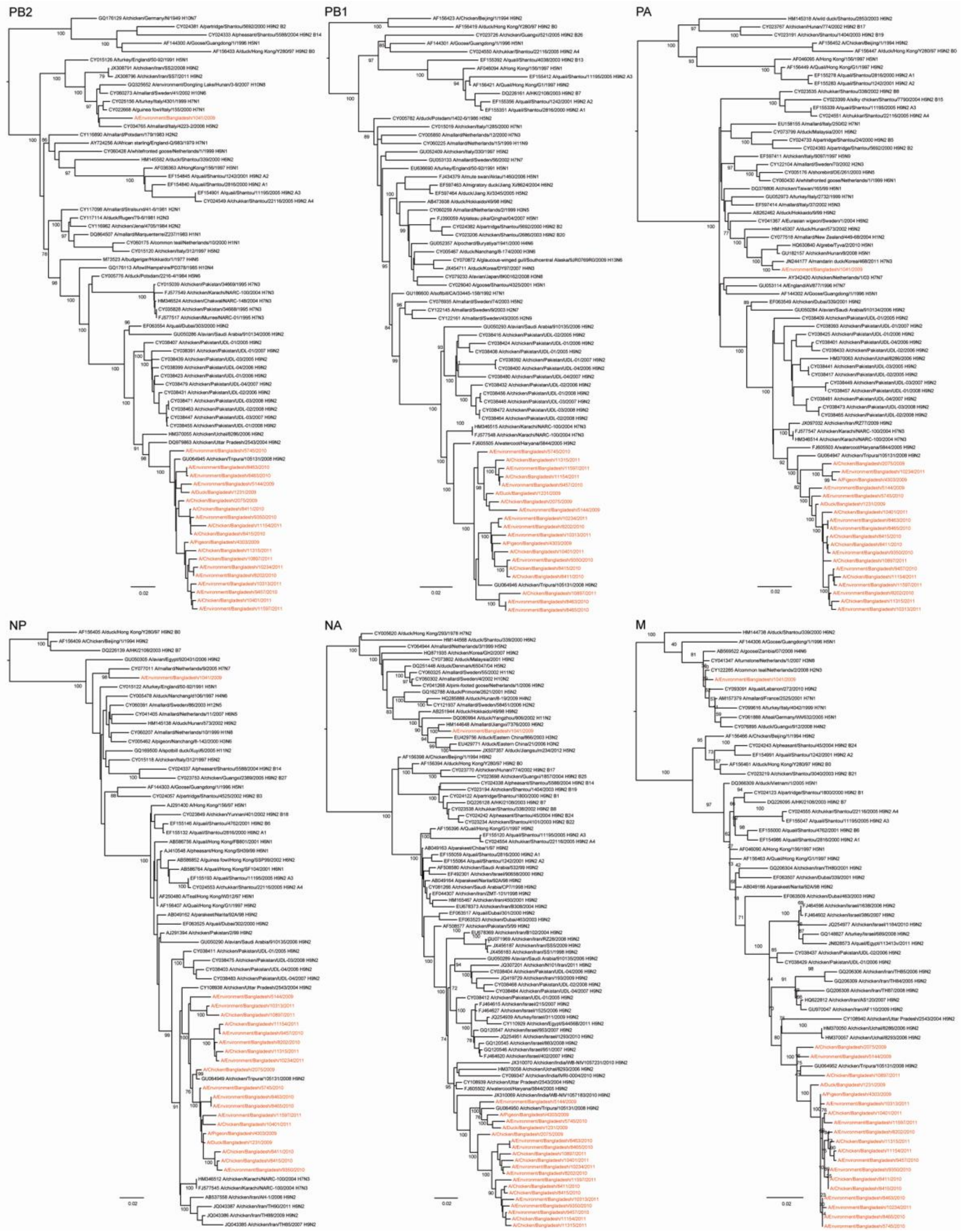

Technical Appendix Figure. Phylogenetic relations of key internal genes of influenza A(H9N2) viruses from Bangladesh. Full-length sequencing, starting from the first codon, was used. The phylogenetic trees were generated by PhyML within the maximum-likelihood framework. Numbers above the branches indicate bootstrap value, and only values greater than 60 are shown. Influenza A(H9N2) viruses from Bangladesh are shown in red.
